# Gastrointestinal perforation in anti-NXP2 antibody-associated juvenile dermatomyositis: case reports and a review of the literature

**DOI:** 10.1186/s12969-020-00486-x

**Published:** 2021-01-06

**Authors:** Yingjie Xu, Xiaolin Ma, Zhixuan Zhou, Jianguo Li, Jun Hou, Jia Zhu, Min Kang, Jianming Lai, Xiaohui Li

**Affiliations:** 1 Department of Rheumatology, Capital Institute of Pediatrics-Peking University Teaching Hospital, 2 Yabao Road, Beijing, China; 2 Department of Cardiology, Capital Institute of Pediatrics-Peking University Teaching Hospital, 2 Yabao Road, Beijing, China

**Keywords:** Juvenile dermatomyositis, Gastrointestinal perforation, Anti-NXP2 antibody

## Abstract

**Background:**

To summarize the characteristics of gastrointestinal (GI) perforation in anti-nuclear matrix protein 2 (NXP2) antibody-associated juvenile dermatomyositis (JDM).

**Methods:**

Five patients with GI perforation from a JDM cohort of 120 cases are described. Relevant literature was reviewed.

**Results:**

Five patients, including four females and one male, were included in the study. The age of onset of these patients ranged from 3.3 to 9.5 years with the median age of 5.0 years. When these patients were complicated by GI perforation, childhood myositis assessment score (CMAS) ranged from 1 to 5 with the median score of 2. Myositis-specific antibody (MSA) spectrum analysis indicated that the five patients were anti-NXP2 antibody positive. The initial symptoms of GI perforation were progressive abdominal pain and intermittent fever. Two patients also presented with ureteral calculus with hydronephrosis and ureteral stricture. Surgery was performed in four patients. One patient failed to undergo a repair as the perforation was high in position. For the other three patients, perforation repair was successful, of which two patients failed due to recurrent perforation. At 24 months postoperative follow-up, one patient was in complete remission on prednisone (Pred) and methotrexate (MTX) treatment, and her ureteral stricture had disappeared. The other four patients died. Adding these cases with 16 other patients described in the literature, the symptom at onset was progressive abdominal pain, which often occurred within 10 months after JDM was diagnosed. Perforation most commonly occurred in the duodenum, although it also occurred at multiple sites or was recurrent. The mortality rate of GI perforation in JDM was 38% (8/21).

**Conclusions:**

All the five perforation cases in our study subjected to MSA analysis were anti-NXP2 antibody positive. The symptom at onset was abdominal pain. The most common site of perforation was the duodenum in the retroperitoneum, and the lack of acute abdominal manifestations prevented early diagnosis. GI perforation may be a fatal complication in JDM, and early diagnosis is very important. More research is needed to determine the pathogenesis and predictive factors of GI perforation in JDM.

## Background

Juvenile dermatomyositis (JDM) is an autoimmune disease characterized by proximal myopathy and a characteristic rash. However, multiple systems, including the digestive system, can also be involved. The occurrence of gastrointestinal (GI) perforation is rare, but a high mortality rate has been reported due to atypical symptoms and difficulty in early diagnosis [1]. Myositis-specific autoantibodies (MSAs) are increasingly used to delineate distinct subgroups of JDM [2, 3]. The presence of anti-nuclear matrix protein 2 (NXP2) autoantibodies substantially increases the risk of calcinosis across all ages and is associated with disease severity [4]. The role of MSAs in cases of JDM complicated by GI perforation has not been reported. Here, from among 120 patients with JDM, we report five cases complicated by GI perforation. All five patients had anti-NXP2 antibody-associated JDM. In addition, we reviewed the other JDM cases with GI perforation reported in the literature to improve our recognition of this disease. Anti-NXP2 autoantibodies may facilitate early diagnosis of the disease.

## Methods

We described five patients with GI perforation from a JDM cohort who were admitted to the Children’s Hospital affiliated with the Capital Institute of Pediatrics, China, from January 2016 to December 2019. The inclusion criteria were: (1) age < 18 years old; (2) diagnosis of JDM based on the Bohan and Peter criteria for myositis [5]; and (3) GI perforation. Patients with other diseases that cause weakness or rash, or a clear alternative diagnosis were excluded from the study. Sixteen MSAs (MDA-5, NXP2, Jo-1, PM-SCL100, PL-7, PL-12, EJ, OJ, anti-Ro52, Ku, PM-Scl, SRP, SAE1, TIF1γ, Mi-2α and Mi-2β) were tested by immune dot blot hybridization using a commercial kit based on goat anti-human IgG antibodies (BlueDot Myositis 12 IgG, #MYO12D-24, D-TEK, Belgium). The study was approved by the Ethics Committee of Capital Institute of Pediatrics. We searched PubMed, Medline and Scopus using the keywords “juvenile dermatomyositis”, “gastrointestinal perforation”, and “perforation”. These literatures with detailed cases which clearly diagnosed JDM and excluded Degos-like presentation with GI perforation were reviewed.

## Results (Table 1)

**Table 1:** Clinical data of JDM patients with GI perforation

Patient /Year	Gender	Age at onset (y)	CMAS score	CAT-A score	CAT-D score	Positive for MSA	Treatment before GI perforation	Symptoms of GI perforation	Duration from onset to perforation (m)	Site of perforation	Surgical treatment	Histopathology of perforation site	Medical Treatment	Follow-up(m) /Outcome
P1	F	3.4	5	2	3	Anti-NXP2	Pred+MTX + IVIG	Abdominal pain/ bloody stools	4/9	Duodenum/ colon	Transverse colon ostomyv duodenostomy	Inflammatory cells infiltrate/ No vasculitis	Pred+Mp pulse+CsA+ IVIG→Pred+Thal+CYC→ Pred+CYC (Po) + MTX → Pred+MTX	24/Remission
P2	F	5.0	1	7	5	Anti-NXP2	MP + MP Pulse+CYC HCQ/MP + MP Pulse+MTX	Abdominal pain/ bloody stools	10	Duodenum/ Duodenal bulb hepatic artery rupture	Vessel sutured/ placement of duodenal jejunal tube (failed)	ND	Pred+MP Pulse+CYC+ IVIG	Death/Infection
P3	M	3.3	5	6	5	Anti-NXP2	Pred+MTX + IVIG/ Pred+IVIG +RTX	Abdominal pain and distention bloody stools	10	Unknown	No surgery	ND	MP pulse+CYC + IVIG	Death/Abdominal bleeding
P4	F	6	2	6	6	Anti-NXP2	Pred+ MTX/CsA →MP pulse+IVIG+CYC	Abdominal pain/ bloody stools	13	Duodenum×2	Perforation repair×2	ND	CYC → MP pulse +IVIG+PE	Death/Shock
P5	F	9.5	2	6	4	Anti-NXP2	Pred+ MTX/CsA	Abdominal pain/ fever and bloody stools	6	Duodenum×2	Perforation repair	ND	MP pulse+ RTX	Death/Abandon treatment
P6 [6] /2016	F	7.0	ND	ND	ND	ND	Pred+MTX	Abdominal pain vomiting and fever	6	Duodenal retroperitoneum	Perforation repair	No vasculitis	ND	ND/Improved
P7 [7] /1997	M	7.0	ND	ND	ND	ND	Pred	Abdominal pain /vomit	24	Duodenum Jejunum	Perforation repair	ND	PE Pred+MP pulse+CYC	12/Stable
P8 [8] /1988	F	5.0	ND	ND	ND	ND	Pred	Abdominal pain	3	Colon	Perforation repair	ND	ND	12/Stable
P9 [8] /1988	F	7.0	ND	ND	ND	ND	Pred +MTX	Abdominal pain	6	The third duodenum	ND	ND	ND	3/ND
P10 [8] /1988	M	8.0	ND	ND	ND	ND	Pred+MTX	Abdominal pain/ fever	6	Esophagus/ duodenum/ colon Mesenteric arterial rupture	Perforation repair	ND	ND	ND/Improved
P11 [8] /1988	F	6.0	ND	ND	ND	ND	Pred+MTX	Abdominal pain and distention/fever	5	Duodenum	Perforation repair	ND	ND	Death/unknown
P12 [9] /2001	M	4.0	ND	ND	ND	ND	Pred+AZA	Abdominal pain/ vomiting/fever	2	Duodenum	Perforation repair	Mucosal ischemia	Pred+MP pulse	19/ND
P13 [10] /2014	M	2.0	ND	ND	ND	ND	ND	Abdominal pain	108	Unknown	ND	ND	ND	Death/unknown
P14 [11] /1985	F	4.0	ND	ND	ND	ND	ND	ND	48	Gastric pylorus	Partial gastrectomy and gastrojejunostomy	ND	ND	ND/Stable.
P15 [11] /1985	F	5.0	ND	ND	ND	ND	ND	ND	3	Middle transverse colon	Loop transverse colon fistula	ND	ND	Death/ARDS
P16 [11] /1985	F	6.0	ND	ND	ND	ND	Pred+MTX/Pred+AZA/ Pre + CYC	Abdominal pain/ vomiting/fever	10	Duodenum colon transverse colon	Multiple perforation repair	ND	ND	7/Stable.
P17 [11] /1985	M	9.0	ND	ND	ND	ND	Pred/Pred+MTX/ Pred+AZA	Abdominal pain/ fever	13	Duodenum	Perforation repair	ND	ND	ND/Stable.
P18 [12] /1984	M	7.0	ND	ND	ND	ND	Pred+MTX	Abdominal pain	10	Duodenal perforation/ colon perforations	Perforation repair	ND	ND	48/Stable.
P19 [13] /2019	F	6.0	ND	ND	ND	ND	Pred+MTX	Abdominal pain/fever	1	transverse colon,	Perforation repair and loop colostomy.	No vasculitis and vasculopathy/ CMV colitis	Steroid+IVIG+Ganciclovir	12/Remission
P20 [14] /2020	ND	10.5	16	ND	ND	ND	ND	Abdominal pain/ GI hemorrhage/ severe bowel obstruction	8/20	Duodenal Perforation/ Jejunal perforation	Jejunal resection/ exclusive parenteral nutrition/ antibiotics,	ND	Steroid+CsA	108/Remission
P21 [14] /2020	ND	10.7	5	ND	ND	ND	ND	Abdominal pain/ severe bowel obstruction	7/9	Esophageal perforation/ Cecal perforation	Colostomy	ND	Steroid+ RTX+ PE	84/Death/Hepatitis

*F* is female, *M* is male, *ND* is no data, *y* is year, *CMAS* is child myositis assessment score, *MSA* is myositis-specific antibody, *m* is month, *Pred* is prednisone, *CYC i*s cyclophosphamide, *IVIG* is intravenous immunoglobulin, *MTX* is methotrexate, *MP* is methylprednisol, *CsA* is cyclosporine A, *RTX* is rituximab, *AZA* is azathioprine, *Thal* is thalidomide, *HCQ* is hydroxychloroquine, *Po* is peros, *CMV* is Cytomegalovirus, *PE* is plasma exchanges, *ARDS* is acute respiratory distresssyndrome, *CAT-A* is cutaneous assessment tool activity, *CAT-D* is cutaneous assessment tool damage

### The general data of the 5 patients

Five patients with GI perforation were identified from 120 cases in a JDM cohort (4.17%). Four of these were females, with the age at onset ranging from 3.3 to 9.5 years (median age − 5.0 years). All five patients showed severe rashes, skin ulcers, and weakness of the skeletal muscles (ranging from power 3/5 to 1/5). When these patients were complicated by GI perforation, childhood myositis assessment score (CMAS) ranged from 1 to 5 with the median score of 2, cutaneous assessment tool (CAT) activity score ranged from 2 to 7 with the median score of 6, CAT damage score ranged from 3 to 6 with the median score of 5. No calcification was observed. Two patients also presented with ureteral calculus with hydronephrosis and ureteral stricture. Analysis of MSAs showed that the five patients were anti-NXP2 antibody positive. There are 31 patients with anti-NXP2 antibody positive in the cohort. The incidence of GI perforation in anti-NXP2 antibody positive patients in our study was 16.13% (5/31). One patient had increased level of cytomegalovirus (CMV)-DNA copies in blood. GI perforation occurred from 4 to 13 months after JDM was diagnosed. The symptoms at onset were severe abdominal pain, fever, GI bleeding and vomiting. Perforations occurred in the duodenum (3 patients), duodenum and colon (1 patient), and an unclear location (1 patient), and perforations at multiple sites or at recurrent times occurred in four patients. All patients were treated with corticosteroids and immunosuppressant agents [cyclophosphamide (CYC)/methotrexate (MTX)/cyclosporine A (CsA)]. One patient was treated with plasma exchange (PE) therapy, and two patients received rituximab (RTX). All five patients were given proton-pump inhibitors (omeprazole). Surgery was performed in four patients, of which one patient failed to undergo repair due to the high position of perforation. For the other three patients, perforation repair was successful, of which two patients failed due to recurrent perforation. At 24 months postoperative follow-up, one patient was in complete remission under Pred and MTX treatment, and her ureteral stricture had disappeared. The other four patients died.

#### Case 1

A 3.4-year-old girl, suffering from characteristic skin rash and muscle weakness was diagnosed with JDM in a local hospital and was treated with Pred(2 mg/kg/d) and MTX (12 mg/m^2^/wk). Muscle weakness improved after 3 months of treatment, following which the dosage of Pred was reduced. When Pred was reduced to 10 mg/d, the girl developed infection in the lungs, skin ulcers around the anus and abdominal pain. One month later, a perforation in the transverse colon was confirmed, and colostomy was performed in a local hospital. She received Pred, CYC, intravenous immunoglobulin (IVIG) and MTX, after which her abdominal pain disappeared, and her muscle strength improved. Seven months after JDM diagnosis, ureteral calculus with hydronephrosis and ureteral stricture were diagnosed and treated via ureteroscopy. Nine months after JDM diagnosis, she developed severe abdominal pain and worsening muscle weakness, for which pulse methylprednisolone (MP) and CsA were given. Later, GI bleeding and fever recurred. She was transferred to our emergency department. She had shock, and her contrast-enhanced computed tomography (CT) abdomen indicated encapsulated effusion and pneumatosis, duodenal perforation and an abscess, which were confirmed during surgery. After duodenostomy, Pred, pulse CYC and thalidomide were given, and when her condition stabilized, we switched to oral Pred, CYC and MTX. At 24 months postoperative follow-up, she was in complete remission with Pred and MTX treatment, and her ureteral stricture had disappeared.

#### Case 2

A 5-year-old girl suffering from characteristic skin rash and muscle weakness was diagnosed with JDM in a local hospital and treated with Pred, MTX and hydroxychloroquine (HCQ), after which her muscle strength improved. But her parents stopped the treatment. Twelve months after JDM diagnosis, she was admitted to our emergency department due to abdominal pain, vomiting, and dark coloured stools. Physical examination showed a poor mental response, painful expression, severe malnutrition, High spring sign (+), partially scabbed skin ulcers in the lower jaw, popliteal fossa and axillae and muscle weakness. A contrast-enhanced CT abdomen indicated peritonitis, intestinal perforation and a right renal-ureteral calculus with pelvicalyceal-ureteral dilation. During surgery, multiple perforations of the duodenum and ruptured right hepatic artery were found. The ruptured blood vessel was sutured, but duodeno jejunal tube placement was unsuccessful. The patient subsequently died of infections and haemorrhagic shock.

#### Case 3

**Fig. 1:**
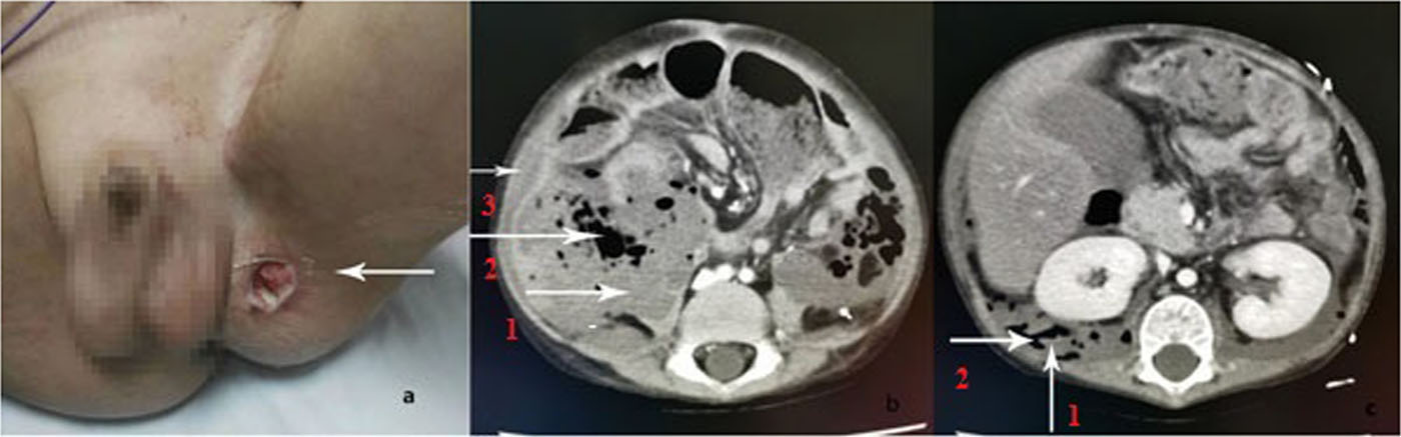
**a** The thigh skin ulcers accompanied by swelling of the ipsilateral testis on Case 3(arrow) **b.** Contrast-enhanced CT showed retroperitoneal effusion (arrow 1), retroperitoneal gas (arrow 2) and abdominal wall hemorrhage (arrow 3) on Case 3. **c** Contrast-enhanced CT showed retroperitoneal effusion (arrow 1) and retroperitoneal gas (arrow 2) on Case 3

A 3.3-year-old boy suffering from characteristic skin rash and muscle weakness was diagnosed with JDM and treated with Pred, MTX and IVIG in a local hospital. His symptoms improved after treatment; the dosage of Pred was reduced, and MTX was stopped due to cataracts and abnormal liver function. The rash and muscle weakness worsened. Nine months after JDM diagnosis, physical examination revealed that severe proximal muscle weakness (power 3/5) and numerous purplish red rashes and ulcerative scabs which could be seen on the face and limbs. MP, RTX and IVIG were administered in our hospital. Two weeks later, he gradually developed abdominal pain, bloating, low back pain, right lower quadrant tenderness and rebound pain. Localized colitis was suspected based on a CT scan. His abdominal pain and distension were relieved after the administration of antibiotics, MP (20 mg/kg/d for 2 days) and IVIG. Four days later, he developed fever, abdominal pain and distension, and an ultrasound showed echoic enhancement in the omentum of the right lower quadrant with weak peristalsis and ascites. MP, pulse CYC and IVIG were continued, and his temperature returned to normal with improved abdominal pain. However, 7 days later, he experienced severe abdominal pain and distension with a rapid decrease in his haemoglobin (Hb) level (from 112 g/L to 67 g/L). Ultrasound showed abdominal muscle inflammation with haemorrhage, and contrast-enhanced CT showed generalized retroperitoneal infection with abdominal wall haemorrhage and intestinal perforation (Fig. 1). Surgical intervention was not possible in view of unstable vital signs, severe infection, and severe and extensive intestinal lesions that could not be surgically repaired. Repeated blood transfusions, anti-microbial agents, and treatment for the primary disease were given. He persisted to have abdominal pain and bloating. A slight change in body position caused massive abdominal wall bleeding. He died after returning home.

#### Case 4

**Fig.2:**
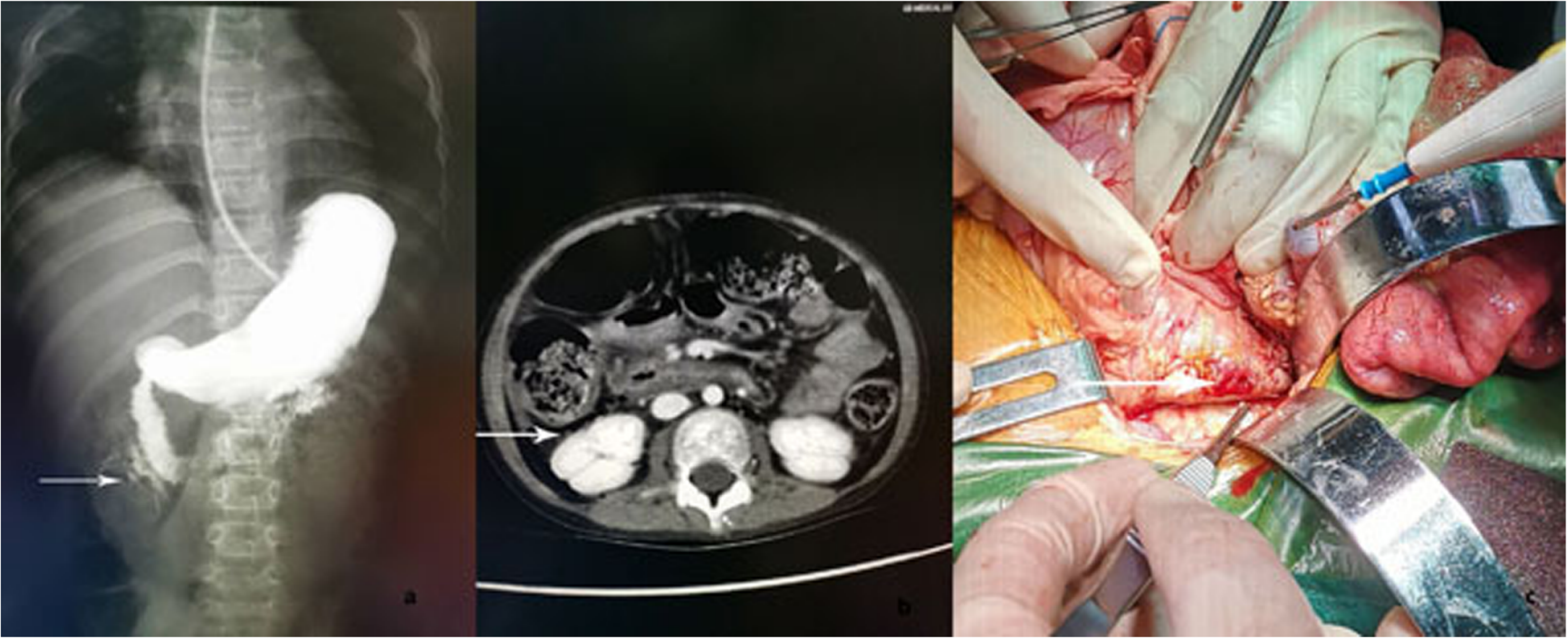
**a** Contrast leakage seen at the junction of the descending and horizontal segments of the duodenum on Case 4 (arrow). **b** Contrast-enhanced CT showed retroperitoneal gas on Case 4 (arrow). **c** Duodenal perforation can be seen at surgery on Case 4 (arrow)

A 7-year-old girl with a past history of JDM was treated 1 year earlier with MP and an immunosuppressant. She presented with progressive proximal muscle weakness, skin ulcers and intermittent abdominal pain for 1 month. Physical examination showed severe malnutrition, anemia, maculopapular rashes symmetrically distributed over the extensor sides of the joints of all extremities, partially scabbing skin ulcers on the extensor side of the left elbow and in both axilla, proximal muscle weakness and edema in both upper limbs. Her Hb level was 59 g/L. Abdominal contrast-enhanced CT did not indicate obvious abnormalities. Ultrasound of the abdomen suggested thickening of the abdominal bowel wall and poor bowel motility. After treatment with blood transfusion, MP and CYC were given. Her symptoms improved, and abdominal pain disappeared. Hb decreased again after 2 weeks, and fever and abdominal pain reappeared again after 4 weeks. Abdominal CT revealed bowel perforation. She immediately underwent surgical repair (Fig. 2). One week later, abdominal pain appeared again. Abdominal CT showed another intestinal perforation. Surgical repair and PE therapy were performed. Her abdominal pain was relieved, but she developed severe pulmonary infection. Three days later, she had manifestations of peritonitis and then developed seizures. Her blood pressure was normal, cerebrospinal fluid tests were negative, but her magnetic resonance imaging (MRI) brain showed that the right temporal lobe and bilateral parieto-occipital lobes were swollen with abnormal signals; patchy abnormal signals were observed in the right thalamus, showing long T2 signal, high T2 FLAIR signal, limited dispersion, and reduced ADC value. We speculated primary disease involving the nervous system. Seizures did not recur after treatment of the primary disease. A repeat MRI brain was not done. Pulse MP and anti-microbial agents were given. Surgical intervention was not possible. The patient died due to uncontrolled bleeding and refractory shock.

#### Case 5

A 10-year-old girl had been diagnosed with JDM 6 months earlier and received Pred and immunosuppressants. She presented with intermittent abdominal pain and fever for 1 month. She was initially treated with pulse MP and IVIG. But her muscle weakness and rash did not improve. She was admitted to our hospital with GI haemorrhage for 3 days. Physical examination showed a poor mental response, painful expression, severe malnutrition, anemia, scattered maculopapular rashes and ulcers distributed in the neck, axillae, buttocks, and extendible joints, and severe proximal muscle weakness. Her Hb level was 54 g/L. Ultrasound of the abdomen suggested thickening of the abdominal bowel wall and poor bowel movements. Pulse RTX and MP were given. Three days later, her abdominal pain worsened. Ultrasound of the abdomen showed intestinal perforation. During surgery, a 5-cm perforation of the transverse part of the duodenum was observed, the perforation was repaired and a double cavity jejunal fistula was performed. Her abdominal pain was relieved. CMV-DNA was detected in the blood, and she was given ganciclovir treatment. Four days later, she again experienced abdominal pain and muscle spasms. Considering the necrosis and perforation of the lower part of the duodenum, her parents withdrew treatment, and the patient died.

### Results of the literature review

Sixteen patients of JDM with GI perforation were identified in PubMed, Medline and Scopus [6–18] which were published between 1984 and 2020. Eight patients were female (50%), six patients were male (37.5%), and the sex of two patients was not recorded (12.5%). The age of onset of these patients ranged from 2 to 10.5 years with the median age of 6.5 years. No clear MSA results were recorded in any case. Primary treatment with Pred and immunosuppressants did not have a significant effect. GI symptoms occurred from one to 108 months after treatment, most of which occurred within 10 months. The initial GI symptoms were severe abdominal pain with fever or vomiting. Perforations were reported in the duodenum (10/16, 62.5%), colon (6/16, 37.5%), jejunum (2/16, 12.5%), oesophagus (2/16, 12.5%), gastric pylorus (1/16, 6.25%), caecum (1/16, 6.25%) and an unclear location (1/16, 6.25%); perforations at multiple sites or recurrent perforations were reported in six patients (6/16, 37.5%). Only five patients had records regarding treatment of the initial disease, and they were treated with Pred or MP and an immunosuppressant (CYC/CsA). One patient underwent PE therapy, one patient received PE therapy with RTX and surgery was performed in 14 patients. In particular, one patient was treated with ganciclovir for CMV infection. Four patients died (25%), and the others were discharged from the hospital after they experienced relief of their symptoms. The clinical data of all 16 patients taken from the literature and the five patients in our study is summarized in Table 1.

## Discussion

JDM, the most common inflammatory myopathy of childhood, is a rare systemic autoimmune vasculopathy that is characterized by weakness in proximal muscles and pathognomonic skin rashes [19]. Multiple systems can be involved, but GI perforations in anti-NXP2 antibody-associated JDM are rarely reported. From among 120 patients with JDM, we report five anti-NXP2 antibody-associated JDM complicated by GI perforation. Most patients in our study were females, with a proportion that was significantly higher than that for the other patients reported in the literature. At the age of onset of these five patients ranged from 3.3 to 9.5 years with the median age of 5.0 years, and GI perforations appeared between 4 and 13 months post diagnosis, which was similar to the 16 cases reported in the literature. When our patients were complicated by GI perforation, CMAS were very low, CAT activity score indicated moderate activity, which suggest that GI perforations may be associated with disease severity and activity. The initial symptoms of JDM with GI perforation were severe abdominal pain, abdominal distention, vomiting, fever and bloody stools. Early identification of perforation was very difficult. With regard to patient (P) 1 and P2, the doctors did not recognize that GI perforation had occurred after persistent abdominal pain for more than 1 month. The perforation in P3 occurred during hospitalization in our hospital and manifested as severe abdominal pain and distension with decreased bowel sounds. Repeated abdominal ultrasounds, X-rays and contrast-enhanced CTs indicated the presence of a low-signal mass in the right lower quadrant with bowel distension, thinning of the intestinal wall and local colitis without pneumoperitoneum or subphrenic pneumatosis. Transitory relief of symptoms occurred after treatment, but severe abdominal pain quickly reappeared, and contrast-enhanced CT and ultrasound showed intestinal perforation, diffuse abdominal wall bleeding and infection. We did not know the precise time when the perforation occurred, suggesting that once abdominal pain appears, perforation should be strongly suspected, and imaging should be performed. Perforation should be considered when abdominal pain is not relieved by active treatment in patients with JDM, and it can be diagnosed early with contrast-enhanced CT scans [6]. We also suggest that repeated scanning and GI angiograms are essential, if the diagnosis cannot be confirmed during the first scan. In the 16 patients in the literature, perforation often occurred in the duodenum (partially in the retroperitoneal area in P6) and the colon. In addition, multi-site or recurrent perforations were also observed in some patients (P7,P10,P16,P18,P20,P21), similar to the results of our study. Furthermore, arterial rupture near the perforation site often occurs, as observed in P2, who had ruptured hepatic arteries, and in P10, who had a ruptured duodenal proximal mesenteric artery; therefore, haemorrhage may also be considered when an unexplained decrease in the Hb level occurs in JDM patients. In addition, several patients have been described in literature with Degos-like lesions linked to GI perforation [20, 21]. A similar deposit complex and pattern of vasculopathy has been found in patients suffering from Degos disease and JDM [22]. We should pay attention to the identification of Degos-like disease. Degos - like skin lesions were initially pale red papules followed by enlarged lesions, showing a characteristic pitting center with a concave navel porcelain white center and a red halo around the periphery. After the rash subsided, there was a white superficial scar. The rash is more common on the trunk and extremities, less common on the palms and feet, and often occurs in batches, old and new. The appearance and location of the rash in five patients of our study were different from Degos-like disease. Further hisopathological identification may be performed if necessary.

In addition, two patients (P1, P2) had urinary system involvement in this group of cases, including ureteral stones, uretero-pelvic dilation and ureteral stenosis. With remission of the primary disease, ureteral stenosis disappeared in P1. This leads us to speculate that ureteral stenosis may be related to vasculopathy involving the urinary system. Morita et al. [23] reported a case of dermatomyositis who developed duodenal perforation as well as ureteral stenosis, with biopsy of stenotic ureter showing calcification with granulomatous tissue without any vasculitic lesion. Ruby Haviv et al. [24] reported a case of juvenile polymyositis (JPM) with right ureteral obstruction secondary to necrosis. The histopathology report of the resected ureter revealed moderate, acute, and chronic inflammatory infiltrates within the ureter wall, urothelial erosions, superficial granulation tissue, and degenerated smooth muscle fibres within the muscularis propria; some of which were infiltrated with lymphocytes and plasma cells, and multiple calcifications within the lamina propria. It also showed fragments of gross calcifications, similar calcifications in other parts of the ureter and obstructing calcifications within small blood vessels. Ruby Haviv et al. considered calcified ureteral necrosis to be a feature of visceral vasculopathy, related to both JDM and JPM. The mechanism is obscure, and the presence of calcifications within visceral tissues might suggest the pathogenesis of the typical calcinosis related to these diseases [24]. Considering that NXP-2 is a type prone to calcification, it was not clear whether ureteral calculi and ureteral stenosis in P1 and P2 were related to vasculopathy with calcification, which needs further studies of visceral calcinosis.

The underlying mechanisms of GI perforation in JDM are still obscure. Some researchers have reported glucocorticoids, non-steroidal anti inflammatory drugs and MTX to be risk factors for GI ulceration and perforation, especially at 1 month post-treatment or with an accumulated dose of steroids higher than 1 g [17]. In our study, Pred was reduced to a small dose before GI symptoms appeared in P1. After perforation was identified, pulse MP pulse and CYC were given, and the patient’s condition improved; thus, the perforation was not attributed to drug factors. Schneider et al. [6] assumed that ulceration in the proximal region and bulb of the duodenum was related to medication, while perforation in the descending part was related to vasculitis, causing intestinal ischemia and necrosis due to thrombogenesis and vascular occlusion. Recent researchers [16, 17] reported that perforated sites showed chronic vasculopathy rather than acute vasculitis. This chronic vasculopathy is characterized by narrowing or complete occlusion of multiple small and medium arteries, subintimal foam cells, fibromyxoid neointimal expansion, and significant luminal compromise with infiltration of macrophages through the muscle layers into the intima. Histopathology at the perforation site was recorded in three patients (P6, P12, P19) in the literature [6, 9, 13], and no vasculitis was noted. P12 exhibited mucosal ischemia, and P19 exhibited vasculopathy and CMV colitis. No manifestation of vasculitis was observed in the histopathology of the fistula. Histopathology of perforation site in P1 of our study showed inflammatory cells infiltrate and no vasculitic lesion was observed in the tissue. The other three patients were unable to undergo histopathology due to erosion of the perforation site. Therefore, whether chronic non-inflammatory vasculopathy causes ischemia and perforation must be considered. Bhaskaran et al. [13] reported a case of intestinal perforation in a child with JDM caused by CMV colitis. CMV-DNA was also detected in the blood of P5. No relevant pathological tests were performed. The other four patients did not undergo blood CMV-DNA tests. The role of CMV colitis in perforation requires further study.

The 120 patients in the JDM cohort were all tested by MSAs analysis, all five patients with GI perforation had strong positivity for anti-NXP2 antibody (3+), and no GI perforation was noted in other MSA patients, which led us question whether anti-NXP2 antibody is associated with GI perforation. After reviewing 16 cases in the literature, Besnard et al. [13] reported that most patients with severe GI manifestations were positive for anti-NXP2 antibody or anti-TIF-1γ antibody. Unfortunately, no specific antibody type was recorded in two patients with GI perforation in this study. The other patients described in the literature had not been tested for MSAs. In 2017, Tansley et al. [25] mentioned that 14 children who were positive for anti-NXP2 antibody had ulcers. Aouizerate et al. [26] reported that anti-NXP2 antibody had a positive association with GI involvement in JDM. The study evaluated the clinical, biological, and histological manifestations of 23 JDM patients and performed a multivariate analysis of 26 histopathological parameters. A case-control study can be performed to further uncover the relationship between anti-NXP2 antibody and GI perforation, which may further reveal the pathological mechanism of GI perforation. Based on our experience in this study, or even early in disease, clinicians should be highly alert to the possibility of GI perforation when abdominal pain occurs on anti-NXP2 antibody-positive patients with JDM.

The mortality rate among our patients was higher than that reported in the literature, which might be due to their severe condition and poor response to various therapeutic drugs, delayed surgery, surgical difficulties at the perforation site, economic reasons and the deficient number of cases. When a patient has intermittent abdominal pain and GI ulcers, the primary disease should be treated more actively to prevent GI perforation. Once GI perforation occurs, the primary disease is not well controlled, and the prognosis is poor. We need more research to choose appropriate treatment strategy for these diseases.

## Conclusion

In this study, the clinical details of five cases of anti-NXP2 antibody-associated JDM complicated by GI perforation were summarized for the first time. Based on the these cases combined with a literature review, we suggest that once JDM patients present with abdominal pain, especially anti-NXP2 antibody-positive patients, GI perforation should be considered. For timely diagnosis, dynamic imaging is important. GI perforation may be a fatal complication of JDM, more research is needed to determine the pathogenesis and predictive factors of GI perforation in JDM.

## Data Availability

The datasets during and/or analyzed during the current study available from the corresponding author on reasonable request.

## Abbreviations

JDM: Juvenile dermatomyositis
NXP2: Nuclear matrix protein 2
GI: Gastrointestinal
MSA: Myositis-specific antibody
CMAS: Child myositis assessment score
Pred: Prednisone
CYC: Cyclophosphamide
IVIG: Intravenous immunoglobulin
MTX: Methotrexate
MP: Methylprednisolone
CsA: Cyclosporine A
RTX: Rituximab
AZA: Azathioprine
Thal: Thalidomide
PE: Plasma exchanges
CMV: Cytomegalovirus
CT: Computed tomography
HCQ: Hydroxychloroquine
Hb: Haemoglobin
MRI: Magnetic resonance imaging
JPM: Juvenile polymyositis
CAT: Cutaneous assessment tool
P: Patient
